# The Three *Streptomyces lividans* HtrA-Like Proteases Involved in the Secretion Stress Response Act in a Cooperative Manner

**DOI:** 10.1371/journal.pone.0168112

**Published:** 2016-12-15

**Authors:** Rebeca L. Vicente, Sonia Gullón, Silvia Marín, Rafael P. Mellado

**Affiliations:** Departamento de Biotecnología Microbiana, Centro Nacional de Biotecnología (CNB-CSIC), Madrid, Spain; Uniwersytet Gdanski, POLAND

## Abstract

Overproduction of Sec-proteins in *S*. *lividans* accumulates misfolded proteins outside of the cytoplasmic membrane where the accumulated proteins interfere with the correct functioning of the secretion machinery and with the correct cell functionality, triggering the expression in *S*. *lividans* of a CssRS two-component system which regulates the degradation of the accumulated protein, the so-called secretion stress response. Optimization of secretory protein production via the Sec route requires the identification and characterisation of quality factors involved in this process. The phosphorylated regulator (CssR) interacts with the regulatory regions of three genes encoding three different HtrA-like proteases. Individual mutations in each of these genes render degradation of the misfolded protein inoperative, and propagation in high copy number of any of the three proteases encoding genes results on indiscriminate alpha-amylase degradation. None of the proteases could complement the other two deficiencies and only propagation of each single copy protease gene can restore its own deficiency. The obtained results strongly suggest that the synthesis of the three HtrA-like proteases needs to be properly balanced to ensure the effective degradation of misfolded overproduced secretory proteins and, at the same time, avoid negative effects in the secreted proteins and the secretion machinery. This is particularly relevant when considering the optimisation of *Streptomyces* strains for the overproduction of homologous or heterologous secretory proteins of industrial application.

## Introduction

Most bacterial secretory proteins transported across the membrane via the Sec pathway are released out of the cell in a misfolded manner. The accumulation of these misfolded proteins could interfere with the correct functionality of the cell [1], and triggers a secretion stress response, whereby a so-called CssRS two-component system has been described to activate in *Bacillus subtilis* [2] and *Streptomyces lividans* [3] in order to induce the synthesis of specific proteases, which degrade the misfolded proteins.

The *B*. *subtilis* CssRS two-component system responds to the secretion stress resulting from the overproduction of heterologous extracellular alpha-amylase (AmyQ of *Bacillus amyloliquefaciens*); the phosphorylated regulator CssR activates the synthesis of two HtrA-like proteases (HtrA, HtrB) [4], specifically binding to the *htrA* and *htrB* regulatory regions [5].

The two *B*. *subtilis* HtrA-like proteases have an N-terminal predicted membrane-spanning segment, a catalytic protease domain and a unique C-terminal PDZ domain; which probably participate in binding to the substrate [6]. In spite of the fact that HtrA is a membrane-bound protein, it is also found extracellularly where it acts as a chaperone for YqxI [7]. These combined proteolytic and chaperone activities for HtrA were also previously described for *E*.*coli* HtrA as well [8].

*Streptomyces lividans* has often been used as a host for the secretory production of homologous and heterologous proteins that are valuable for industrial and pharmacological purposes [9]. The optimization of this production is important to maximize the yield and quality of these proteins. Therefore, the characterisation of the control factors involved in the degradation of misfolded proteins is necessary to optimize secretory protein production, avoiding the potential interference of the accumulated misfolded proteins with essential bacterial cell processes.

In our laboratory a CssRS two-component system in *S*. *lividans* was recently identified as being responsible for the degradation of misfolded proteins upon alpha-amylase overproduction and this system surprisingly activates the synthesis of three HtrA-like proteases (HtrA1, HtrA2 and HtrB) [3]. In this work, a direct interaction between CssR and the three proteases encoding genes has been found. The three proteases play an essential role in the degradation of extracellular misfolded proteins. Moreover, the overproduction of these proteases plays a detrimental role in their correct functioning of the secretion stress response in *S*. *lividans*.

## Materials and Methods

### Bacterial strains, plasmids and media

The bacterial strains and plasmids used in this study are listed in Table 1.

**Table 1:** *Streptomyces* bacterial strains and plasmids used in this study.

Strain or plasmid	Relevant characteristics	Source of reference
***S*. *lividans strains***		
TK21	Wild type	John Innes Centre Collection, Norwich UK
*ΔhtrB*	TK21 but *htrB*::pOJ260; *aac(3)IV*	This study
*ΔhtrA1*	TK21 but *htrA1*::pOJ260; *aac(3)IV*	This study
*ΔhtrA2*	TK21 but *htrA2*::pOJ260; *aac(3)IV*	This study
TK21 pIJ487	TK21 carrying plasmid pIJ487; *tsr*	[3]
*ΔhtrB* (pIJ487)	*ΔhtrB* carrying pIJ487; *tsr*,*aac(3)IV*	This study
*ΔhtrA1* (pIJ487)	*ΔhtrA1* carrying pIJ487; *tsr*,*aac(3)IV*	This study
*ΔhtrA2* (pIJ487)	*ΔhtrA2* carrying pIJ487; *tsr*,*aac(3)IV*	This study
TK21 (pAMI11)	TK21 carrying pAMI11	[3]
*ΔhtrB* (pAMI11)	*ΔhtrB* carrying pAMI11; *neo*,*aac(3)IV*	This study
*ΔhtrA1* (pAMI11)	*ΔhtrA1* carrying pAMI11; *neo*,*aac(3)IV*	This study
*ΔhtrA2* (pAMI11)	*ΔhtrA2* carrying pAMI11; *neo*,*aac(3)IV*	This study
TK21 (pAMI11) (pFDT)	TK21 carrying pAMI11 and pFDT; *neo tsr*	This study
TK21 (pAMI11) (pFDB)	TK21 carrying pAMI11 and pFDB; *neo tsr*	This study
TK21 (pAMI11) (pFDA1)	TK21 carrying pAMI11 and pFDA1; *neo tsr*	This study
TK21 (pAMI11) (pFDA2)	TK21 carrying pAMI11 and pFDA2; *neo tsr*	This study
*ΔhtrB* (pAMI11) (pFDT)	*ΔhtrB* carrying pAMI11 and pFDT; *neo tsr aac(3)IV*	This study
*ΔhtrB* (pAMI11) (pFDB)	*ΔhtrB* carrying pAMI11 and pFDB; *neo tsr aac(3)IV*	This study
*ΔhtrB* (pAMI11) (pFDA1)	*ΔhtrB* carrying pAMI11 and pFDA1; *neo tsr aac(3)IV*	This study
*ΔhtrB* (pAMI11) (pFDA2)	*ΔhtrB* carrying pAMI11 and pFDA2; *neo tsr aac(3)IV*	This study
*ΔhtrA1*(pAMI11) (pFDT)	*ΔhtrA1* carrying pAMI11 and pFDT; *neo tsr aac(3)IV*	This study
*ΔhtrA1*(pAMI11) (pFDB)	*ΔhtrA1* carrying pAMI11 and pFDB; *neo tsr aac(3)IV*	This study
*ΔhtrA1*(pAMI11) (pFDA1)	*ΔhtrA1* carrying pAMI11 and pFDA1; *neo tsr aac(3)IV*	This study
*ΔhtrA1*(pAMI11) (pFDA2)	*ΔhtrA1* carrying pAMI11 and pFDA2; *neo tsr aac(3)IV*	This study
*ΔhtrA2*(pAMI11) (pFDT)	*ΔhtrA2* carrying pAMI11 and pFDT; *neo tsr aac(3)IV*	This study
*ΔhtrA2*(pAMI11) (pFDB)	*ΔhtrA2* carrying pAMI11 and pFDB; *neo tsr aac(3)IV*	This study
*ΔhtrA2*(pAMI11) (pFDA1)	*ΔhtrA2* carrying pAMI11 and pFDA1; *neo tsr aac(3)IV*	This study
*ΔhtrA2*(pAMI11) (pFDA2)	*ΔhtrA2* carrying pAMI11 and pFDA2; *neo tsr aac(3)IV*	This study
TK21 (pAMI11) (pSET152tsr)	TK21 carrying pAMI11 and pSET152tsr; *neo tsr*	This study
*ΔhtrB* (pAMI11) (pSETB)	*ΔhtrB* carrying pAMI11 and pSETB; *neo tsr aac(3)IV*	This study
*ΔhtrA1* (pAMI11) (pSETB)	*ΔhtrA1* carrying pAMI11 and pSETB; *neo tsr aac(3)IV*	This study
*ΔhtrA2* (pAMI11) (pSETB)	*ΔhtrA2* carrying pAMI11 and pSETB; *neo tsr aac(3)IV*	This study
*ΔhtrB* (pAMI11) (pSETA1)	*ΔhtrB* carrying pAMI11 and pSETA1; *neo tsr aac(3)IV*	This study
*ΔhtrA1*(pAMI11) (pSETA1)	*ΔhtrA1* carrying pAMI11 and pSETA1; *neo tsr aac(3)IV*	This study
*ΔhtrA2*(pAMI11) (pSETA1)	*ΔhtrA2* carrying pAMI11 and pSETA1; *neo tsr aac(3)IV*	This study
*ΔhtrB* (pAMI11) (pSETA2)	*ΔhtrB* carrying pAMI11 and pSETA2; *neo tsr aac(3)IV*	This study
*ΔhtrA1*(pAMI11) (pSETA2)	*ΔhtrA1* carrying pAMI11 and pSETA2; *neo tsr aac(3)IV*	This study
*ΔhtrA2*(pAMI11) (pSETA2)	*ΔhtrA2* carrying pAMI11 and pSETA2; *neo tsr aac(3)IV*	This study
***Plasmids***		
pUC19	*E*.*coli* cloning vector, *bla*, *lacZα*	[12]
pUC19B	pUC19 derivative containing the *htrB* gene and its regulatory region	This study
pUC19A1	pUC19 derivative containing the *htrA1* gene and its regulatory region	This study
pAC301	pUC18 containing the *tsr* gene; *bla*,	[13]
pGEM-T easy	*E*.*coli* cloning vector, *bla*, *lacZα*	Promega
pET28a	*E*. *coli* expression vector, *bla*	Novagen
pRHIS	pET28a with His-CssR	This study
pOJ260	Bifunctional plasmid *E*. *coli-Streptomyces* used in conjugation experiments; *aac(3)IV*, *lacZα*, *oriT*RK2	[14]
pOJB	pOJ260 derivative containing an *htrB* fragment	This study
pOJA1	pOJ260 derivative containing an *htrA1* fragment	This study
pOJA2	pOJ260 derivative containing an *htrA2* fragment	This study
pFD666	High copy number bifunctional plasmid *E*. *coli*- *Streptomyces*; *neo*	[15]
pIJ486	*Streptomyces* multicopy plasmid containing promotorless *neo*; *tsr*	[16]
pAMI11	pIJ487 derivative containing the *amlB* gene; *neo*	[17]
pFDT	pFD666 containing the *tsr* gene; *neo*	This study
pFDB	pFDT derivative containing *htrB* and its regulatory region	This study
pFDA1	pFDT derivative containing *htrA1* and its regulatory region	This study
pFDA2	pFDT derivative containing *htrA2* and its regulatory region	This study
pSET152	ϕC31-derived integration vector; *aac(3)IV*	[14]
pSET152*tsr*	pSET152 derivative containing the *tsr* gene; *aac(3)IV*	This study
pSETB	pSET152*tsr* derivative containing the *htrB* and its regulatory region; *tsr*, *aac(3)IV*	This study
		This study
pSETA1	pSET152*tsr* derivative containing the *htrA1* and its regulatory region; *tsr*, *aac(3)IV*	This study
pSETA2	pSET152 derivative containing the *htrA2* and its regulatory region and the *tsr* gen; *aac(3)IV*	This study

The *S*. *lividans* TK21 wild-type strain [10] and its derivatives were cultured in liquid NMMP medium using mannitol as carbon source [11]. Apramycin (25 μg/ml), thiostrepton (50 μg/ml), kanamycin (50 μg/ml) and chloramphenicol (25 μg/ml) were added to the R5 and MS solid media, when required.

### Construction of gene disruption mutants

To construct the *htrB* mutant strain, oligonucleotides htrBdis_Fw and htrBdis_Rv (Table 2) were used to amplify a 531 nt DNA fragment from the *S*. *lividans* TK21 genome. To construct the *htrA1* mutant strain, oligonucleotides htrA1dis_Fw and htrA1dis_Rv (Table 2) were used to amplify a 502 nt DNA fragment from the *S*. *lividans* TK21 genome. To construct the *htrA2* mutant strain, oligonucleotides htrAdis2_Fw and htrAdis2_Rv (Table 2) were used to amplify a 675 nt DNA fragment from the *S*. *lividans* TK21 genome. These fragments were inserted into plasmid pOJ260 [14] through its unique *Bam*HI and *Eco*RI sites to generate plasmids pOJB, pOJA1 and pOJA2 respectively. The plasmids were used to conjugate *E*. *coli* to *Streptomyces*, as described [18]. *E*. *coli* ET12567 carrying the non-transmissible ‘‘driver” plasmid pUZ8002 was used for conjugation [19]. Apramycin resistant strains containing the disrupted genes *htrB*, *htrA1* and *htrA2*, respectively, were selected upon verification of the disruption by PCR amplification and Southern blot hybridization analysis (not shown).

**Table 2:** Primer sequences.

Primer pair	Sequence (5’→ 3’)	Target fragment	Restriction site	Product length (bp)
***Proteases mutants***				
htrBdis_FW	GTTGGATCCGGCATCCAGGAGCTGACC	*htrB*	*Bam*HI	531
htrBdis_RV	GGTGAATTCGAACTCGAACGGCCACTG		*Eco*RI	
htrA1dis_FW	GTTGGATCCTGAGCTGGAGGCCGACTAC	*htrA1*	*BamHI*	502
htrA1dis_RV	GGTGAATTCCTGCCCGTCCAGGTTCAC		*EcoRI*	
htrA2dis2_FW	GTTGGATCCCGTACCTGGAACGGAACG	*htrA2*	*BamHI*	675
htrA2dis2_RV	GGTGAATTCAGCCGAGGCCTATGGAAC		*EcoRI*	
***CssR purification***				
CssRHisFw	GTCGGATCCAGCCCCGCAGAC	*cssR*	*Bam*HI	744
CssRHisRv2	GTTAAGCTTTCACTCGGCGCCG		*Hin*dIII	
***EMSA assays***				
Css RpromFW	GTTCTGCAGTGATCGACATGAACGGCA	P_*cssR*_	*Pst*I	302
CssRpromRV	GGCGGATCCATCAGGATGCGCTGGATCT		*Bam*HI	
htrB_Fw2	GTTGGATCCGAGCGGCTGAAGGTGTTC	P_*htrB*_	*Bam*HI	237
htrBpromRv	GGCAAGCTTGTACGGGTTCGCGTGCTC		*Hin*dIII	
2171prom500Fw	CTTCGACGTGGTGCTGTG	P_*htrA1*_	None	549
2171promRv	AGTGTCCATGGCCCGAGT		None	
5149prom500Fw	GGTCCGTGAACCTGATTGAA	P_*htrA2*_	None	535
5149promRV	GTGCCGTCGGCGGCCGGAA		None	
S/U 3,4 D	GGAGAATTCGTGCTTTCCCCTCACTCGT	P_*degU*_	*Eco*RI	110
PR3,4r	GGGGGATCCACCGTACGTGCG		*Bam*HI	
***Complementation of the proteases mutants***				
*htrB*_Fw2	GTTGGATCCGAGCGGCTGAAGGTGTTC	*htrB* and	*Bam*HI	1233
*htrB*_Rv2	GGTAAGCTTCAGTTGCTCGTCAGTTGCTC	regulatory region	*Hin*dIII	
*htrA1*_FW	GTTGGATCCCTTCGACGTGGTGCTGTG	*htrA1* and	*Bam*HI	1629
*htrA1*_RV	GTTAAGCTTTCACTGCTCGCCGAGCGT	regulatory region	*Hin*dIII	
*htrA2*_FW	GTTGGATCCGGTCCGTGAACCTGATTGAA	*htrA2* and	*Bam*HI	2260
*htrA2*_RV	GTTAAGCTTGAACACCTGAAGCTCCTTGG	regulatory region	*Hin*dIII	

Plasmid pAMI11 [17] is a pIJ486 [16] derivative carrying the *S*. *lividans* gene *amlB* (encoding alpha-amylase B) and a frame-shift-mutated thiostrepton resistance gene; which was used to transform the *S*. *lividans* TK21, *S*. *lividans* Δ*htrB*, *S*. *lividans* Δ*htrA1* and *S*. *lividans* Δ*htrA2* protoplasts. Plasmid pIJ487 was propagated in *S*. *lividans* TK21 and protease mutant strains to generate the corresponding isogenic strains (Table 1)

### Construction of complementation strains

For the multicopy complementation of the mutant strains, gene *htrB*, *htrA1* and *htrA2* with their respective predicted regulatory region were amplified with oligonucleotides htrB_Fw2 and htrB_Rv2, htrA1_Fw and htrA1_Rv, and htrA2_Fw and htrA2_Rv (Table 2), respectively. The chromosomal DNA of *S*. *lividans* TK21 strain was used as a template in all cases. The obtained DNA fragments were subsequently sequenced and digested with *Bam*HI and *Hin*dIII and cloned in the respective sites of the multicopy plasmid pFD666 [15].

An 1845-nt long fragment containing the thiostrepton resistance gene (*tsr*) was retrieved from plasmid pAC301 [13] by *Bgl*II. The fragment was inserted into plasmid pFD666 and its derivatives already containing *htrB*, *htrA1*, *htrA2* through their respective *Bam*HI sites generating plasmids pFDT, pFDB, pFDA1 and pFDA2 respectively.

Plasmids pFDB, pFDA1 and pFDA2 were used to transform the *S*. *lividans* TK21, *htrB*, *htrA1* and *htrA2* mutant strains carrying pAMI11 to generate the strains used in this study (Table 1). Plasmid pFDT was propagated into the *S*.*lividans* TK21, *htrB*, *htrA1* and *htrA2* mutant strains carrying pAMI11 to generate the corresponding isogenic strains (Table 1).

To complement the *htrB* and *htrA* mutant strains with their respective genes in monocopy, two DNA fragments, *Eco*RI-*Hin*dIII blunt-ended 1.2 and 1.6 kb long were retrieved from pFDB and pFDA1 respectively. Each fragment, containing the *htrB* and *htrA1* genes with their respective regulatory regions, was inserted in pUC19 previously linearized with *Eco*RI and *Sma*I to generate pUC19B and pUC19A1, respectively. Plasmids pUC19B and pUC19A1 were restricted with *Eco*RI and *Bam*HI to retrieve intact *htrB* and *htrA1* genes that were transferred to the integrative plasmid pSET152*tsr* digested with *Eco*RI and *Bam*HI to obtain pSETB and pSETA1 respectively. The integrative plasmid, pSET152*tsr* was previously obtained by inserting into pSET152 an *Xba*I-*Bam*HI 1.8 kb long fragment containing the *tsr* gene from pFDA1.

To complement the *htrA2* mutant strain with its own gene, an *Eco*RI-*Hind*III blunt ended 4.1 kb long fragment from pFDA2 containing *htrA2* and the *tsr* gene was inserted in pSET152 digested with *Eco*RI and *Bam*HI blunt-ended to obtain pSETA2.

### Quantitative Real Time PCR (qRT-PCR)

Total RNA was isolated from bacteria growing cultures at the exponential phase of growth using the RNeasy midi Kit (Qiagen). Cell lysates were extracted twice with phenol-chloroform before being loaded onto RNeasy midi columns for RNA purification. DNA, potentially contaminating the RNA preparations, was removed by incubation with RNase-free DNAse (Ambion) and its absence was tested by quantitative real time PCR amplification in the absence of reverse transcriptase. Complementary DNA was synthesised using the High Capacity Archive kit (Applied Biosystems). Quantitative real time PCR (qRT-PCR) was performed using SYBR Green technology as previously described [3]. Three biological samples from the different bacterial cultures were amplified in triplicate in separate PCR reactions. All PCR products were between 50 and 150 bp in length.

A melting curve analysis was conducted after amplification to distinguish the targeted PCR products from the non-targeted ones. The melting curves were obtained by heating at temperatures ranging from 60°C to 95°C at a rate of 0.2°C per sec, with continuous fluorescence scanning. The *hrdB* transcript was carried out as an internal control to quantify the relative expression of the target genes as before [3]. The oligonucleotides used as primers were previously described [3].

### Construction and purification of a six-His-tagged CssR protein

To obtain an N-terminally six-His-tagged CssR protein, the *cssR* open reading frame was amplified by PCR using the primers CssRHisFw and CssRHisRv2 (Table 2). The chromosomal DNA of *S*. *lividans* TK21 strain was used as a template. The product of this reaction was digested with *Bam*HI and *Hin*dIII and cloned into the similarly digested pET28a (+) plasmid (Novagen), yielding plasmid pRHIS.

To induce expression, *Escherichia coli* BL21 (DE3) carrying pRHIS was diluted 1:100 to fresh medium from an overnight culture. At an optical density of 600 nm of 0.5, expression was induced by the addition of 1 mM of IPTG (isopropyl-β-D-thiogalactopyranoside). Cells were harvested after an additional 4 hours of growth and pellets were suspended in 20 ml of lysis buffer (NaH_2_PO_4_/ Na_2_HPO_4_ 50 mM, NaCl 400 mM, DNAase I (0.1 mg ml^-1^) in the presence of one tablet of EDTA-free protease inhibitor cocktail [Roche]) and disrupted by two passages through a French pressure cell at 1000 p.s.i. Soluble and insoluble fractions of the *E*. *coli* lysate were separated by centrifugation at 20,000 x g for 1 hour at 4°C. The soluble protein extract corresponding to the cytoplasmic fraction was loaded onto a chromatography column filled with a Cobalt-containing resin (Talon, Clontech). After loading and washing His_6_-CssR it was subsequently eluted with a buffer containing 150 mM imidazole. Fractions containing CssR were collected and analysed by SDS-PAGE 12% (S1 Fig).

The eluted fractions were dialysed and protein concentration was estimated using the BCA protein assay kit (Pierce), as indicated by the supplier.

When necessary, eluted fractions were concentrated using the Centricon filter (10 kDa cutoff; Millipore).

### Protein identification by nano LC–MS/MS Triple Tof analysis

The samples were subjected to methanol-chloroform precipitation to isolate proteins and remove interfering substances and taken to dryness. The protein extracts were dissolved in 8M Urea / 25mM ammonium bicarbonate solution, reduced by 10mM dithiothreitol (DTT), and alkylated by addition of cysteine-blocking reagent (iodoacetamide). Samples were further diluted and digested with trypsin at an enzyme-to protein ratio of 20:1, at 37°C overnight. All reagents were purchased from Sigma-Aldrich.

The peptide samples were analyzed on a nano liquid chromatography system (Eksigent Technologies nanoLC Ultra 1D plus, AB SCIEX, Foster City, CA) coupled to 5600 Triple TOF mass spectrometer (AB SCIEX, Foster City, CA) with a nanoelectrospray ion source. Samples were injected on a C18 PepMap trap column (5 μm, 100 μm I.D. x 2 cm, Thermo Scientific) at 2 μL/min, in 0.1% formic acid in water, and the trap column was switched on-line to a C18 nanoAcquity BEH analytical column (1.7 μm, 100 Å, 75 μm I.D. x15 cm, Waters). Equilibration was done in mobile phase A (0.1% formic acid in water), and peptide elution was achieved in a 40 min linear gradient from 5%–40% B (0.1% formic acid in acetonitrile) at 250 nL/min. The mass spectrometer operated in data-dependent acquisition mode. For TOF scans, the accumulation time was set to 250 ms, and per cycle, up to 15 precursor ions were monitored.

MS and MS/MS data obtained for each sample were processed using Analyst TF 1.5.1 Software (AB SCIEX, Foster City, CA). Raw data were translated to mascot general file (mgf) format and searched against a database built from sequences in the *Streptomyces lividans* TK24 and *Escherichia coli* BL21 (DE3) [20, 21] at Uniprot Knowledgebase (as of Oct 2016), using an in-house Mascot Server v. 2.4 (Matrix Science, London, U.K.). Search parameters were set as follows: carbamoidomethylcysteine as fixed modification and oxidized methionines as variable one. Peptide mass tolerance was set to 25 ppm and 0.02 Da, in MS and MS/MS mode, respectively and 1 missed cleavage was allowed.

### Electrophoretic Mobility Shift Assay (EMSA)

The oligonucleotides used to PCR amplify the respective regulatory regions of the *cssRS* operon, and genes *htrB*, *htrA1*, *htrA2* and *degU* (carried as a negative control) are indicated in Table 2.

The amplified DNA fragments were purified by agarose gel electrophoresis. Purified CssR protein was phosphorylated *in vitro* with acetyl phosphate as described before [22] and was then mixed with the DNA fragments in a 20 μl reaction volume containing 10 mM Tris-HCl pH 8.0, 40 mM KCl, 1 mM MgCl_2_, 2,5 mM dithiothreitol, and 5% glycerol. After incubation at 37°C for 15 minutes and the addition of the DNA dye solution (10% glycerol, 0.02% bromophenol blue), the mixture was loaded directly onto a pre-run 6% polyacrylamide gel. Gel electrophoresis was performed in TBE at 100 V for 2 h at 4°C. After electrophoresis the gel was dyed with a TBE solution containing 0.01% ethidium bromide. Signals were detected with a UV transilluminator (Gel Doc 2000 de BIO-RAD).

### Low resolution DNase I footprinting

A 237-bp long DNA fragment spanning from positions -180 to +57 of the *S*. *lividans htrB* gene was incubated with the phosphorylated CssR, as described above, and subsequently treated with 0.5 units of DNase I (New England Biolabs) at 37°C for 10 min. The reaction was stopped by incubating at 75°C for 10 min. The resultant DNA fragments were extracted with phenol-chloroform and precipitated with ethanol [23]. The DNA fragments were cloned into a *Sma*I digested pUC19 plasmid previously dephosphorylated with alkaline phosphatase from bovine intestine (Roche) and propagated in *E*.*coli* XL1-BLUE. Fourteen pUC19-derivative plasmids were purified and their respective DNA sequenced with an automated DNA sequencer. The resulting sequences were aligned using BLAST and MEME web servers to identify possible protected regions from DNAase digestion.

A putative consensus motif, C(C/G)AGCT(G/T)CG, was derived when the equivalent regions from *cssR*, *htrA1*and *htrA2* were compared to that of *htrB*. This putative motif was used to search the *Streptomyces lividans* genome using RSAT [24] with default settings.

### Protein analysis and Western blot experiments

Supernatants from the HtrA-like proteases deficient strains overproducing alpha-amylase (AmlB) and from HtrA-like proteases deficient strains overproducing the different HtrA-like proteases and the AmlB protein were grown in NMMP medium [25] were processed as described [3]. Intracellular protein analysis was carried out as indicated previously [3]. For Western blot analysis, cell-associated and extracellular proteins were fractionated by SDSPAGE in 10% (w/v) acrylamide gel [26].

*E*. *coli* overexpressing His_6_-CssR cell lysates were fractionated by SDSPAGE in 12% acrylamide gel (w/v). Gel-fractionated proteins were transferred onto immobilon polyvinylidene difluoride membranes (Milipore), as described [27]. To perform Western blot analysis of the AmlB overproducer strains, the transferred material was incubated with polyclonal antibodies raised against *S*. *lividans* TK21 AmlB (a gift from C. Isiegas) followed by incubation with HRP-conjugated protein A (Invitrogen Laboratories) as described before [3]. Transferred His6-CssR proteins fractions were incubated with the monoclonal antibody 6xHis mAb-HRP Conjugate, Clontech (Takara ref. 631210)

### Enzyme activity

To determine extracellular alpha-amylase activity, the supernatants from 20-ml aliquots of bacterial cell cultures at the indicated phases of growth were processed as previously described [3]. The alpha-amylase activity was estimated by determining the amount of reducing sugar released from starch. The assay was carried out by adding supernatant sample and starch solution 1% (w/v) treated with NaBH4, as described [28] in 20 mM phosphate buffer and was incubated at 37°C for 30 min. The reaction was stopped by the addition of dinitrosalicylic acid [29]. One unit of alpha-amylase was defined as the amount of an enzyme necessary to produce reducing sugar equivalent to 1 μmol of glucose in 30 min under the assay conditions. The specific activity, measured as units per mg of protein, was the average of at least three independent determinations. The protein concentration in the different samples was determined using the BCA protein assay kit (Pierce), as indicated by the supplier.

## Results

### CssR interacts with *htrA1*, *htrA2*, *htrB* and *cssRS* regulatory regions

**Fig 1:**
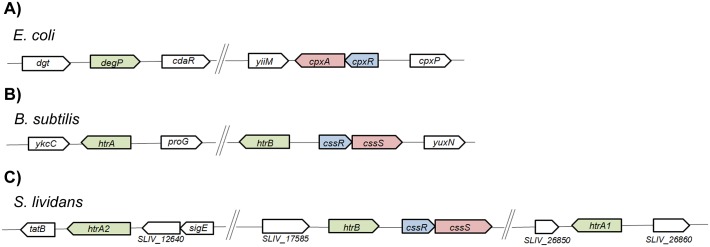
Gene organization of the two-component system and the HtrA-like proteases encoding genes. (A) Schematic representation of the *E*. *coli* chromosomal region of the *cpxRA* two-component system, the gene encoding the *E*. *coli* HtrA-like protease (*degP*) and adjacent genes. Schematic representacion of the *B*.*subtilis* (B) and *S*. *lividans* (C) chromosomal regions of the *cssRS* two-component system, the HtrA-like proteases encoding genes and adjacent genes.

HtrA1, HtrA2 and HtrB, are HtrA-like serine proteases. The *htrB* gene is located immediately upstream of the *cssRS* two-component operon in a similar chromosomal organisation to that of *B*. *subtilis* while *htrA1* and *htrA2* are located far from *cssRS* in the bacterial genome (Fig 1).

The overproduction of alpha-amylase in *S*. *lividans* causes the secretion stress that activates the CssRS two-component system, which apparently regulates *htrA1*, *htrA2* and *htrB* expression [3]. In *E*. *coli* and *B*. *subtilis* the phosphorylated CssR activates the expression of the HtrA-like protease genes and regulates the expression of its own operon [4, 30].

To further investigate if the *S*. *lividans* regulator CssR interacts with the respective *htrA1*, *htrA2*, *htrB* regulatory regions and with that of the *cssRS* operon, EMSA experiments were carried out using purified N-terminal His-tagged CssR protein.

The purified N-terminal His-tagged CssR protein was analysed using nano LC–MS/MS Triple Tof analysis as indicated in Material and methods. The *S*. *lividans* CssR protein has the highest Mascot protein score where 73% of the total identified peptides belonged to the CssR protein. Proteins with a significantly lower Mascot protein score are usually appearing when recombinant His-tagged proteins were expressed in *E*. *coli* and purify by immobilized metal affinity chromatography (IMAC) [31,32]. Additionally, neither of them have a regulatory function (S1 Table).

**Fig 2:**
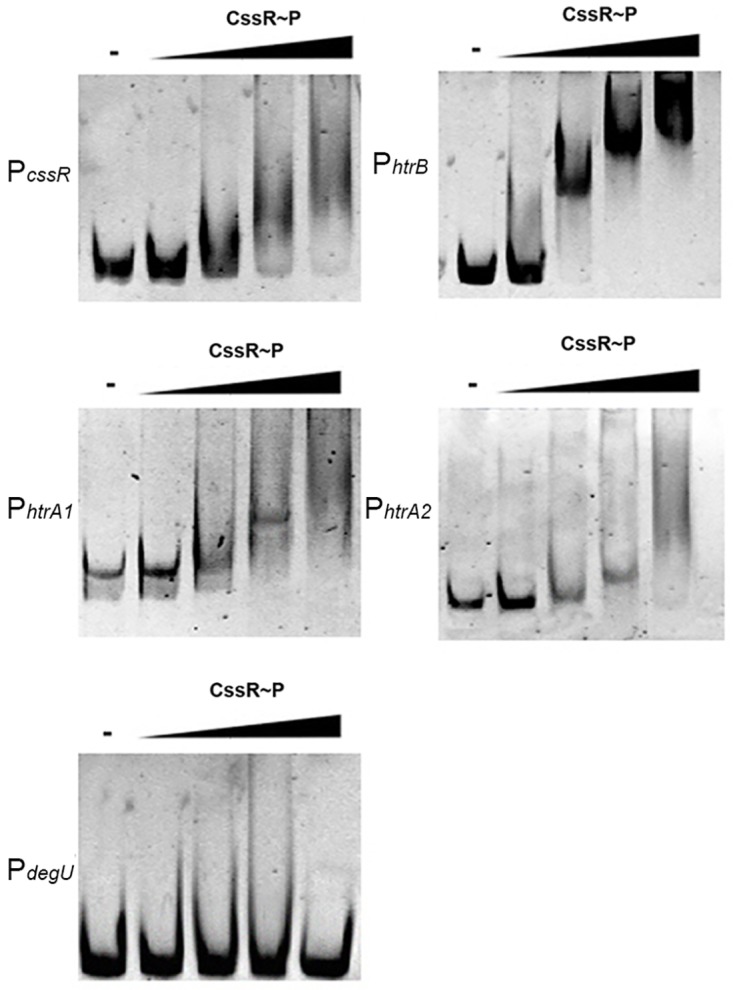
CssR binds to the regulatory regions of the genes encoding the three HtrA-like proteases. DNA fragments containing the respective potential regulatory regions of the three HtrA-like protease genes and the *cssSR* operon were incubated with growing concentration of phosphorylated CssR (0, 4, 13, 26, 44 μM) and subjected to gel shift assay; the *degU* regulatory region was carried out as a negative control.

The 302, 237, 549 and 535 long DNA fragments containing the potential regulatory regions of *cssR*, *htrB*, *htrA1* and *htrA2* respectively, and the 110 bp long fragment containing the regulatory region of *degU*, carried as a negative control, were amplified using the oligonucleotides described in Table 2. His-CssR was labelled in vitro with acetyl phosphate and used in the reaction mixtures to enhance binding the protein to the target sequences [5]. As expected, the phosphorylated six-histidine-tagged CssR retarded the mobility of all the DNA fragments used, except that containing the *degU* regulatory region (Fig 2), confirming that CssR interacts with the regulatory regions of the genes encoding the HtrA-like proteases and with that of the *cssRS* operon. Two smaller DNA fragments 276 and 351 bp long from the respective *htrA1* and *htrA2* potential regulatory regions, were not retarded in the EMSA assays (not shown) and the 549 and 535 bp long fragments were respectively used instead.

Low resolution DNAase I footprinting experiments (see Material and methods) allowed the identification of a putative conserved motif C(C/G)AGCT(G/T)CG, which was absent in the non-retarded DNA fragments.

### The three HtrA-like proteases are equally needed in the secretion stress response

To determine the role of the three proteases in the secretion stress response, individual mutants in each of the respective genes were constructed by disruption to generate *S*. *lividans* Δ*htrB*, *S*. *lividans* Δ*htrA1* and *S*. *lividans* Δ*htrA2* mutant strains. The relative expression levels of *htrB*, *htrA1* and *htrA2* were analysed by quantitative RT-PCR (qRT-PCR) in each mutant strain with respect to that of the wild type strain. Thus, *htrB* (-3.38± 1.04), *htrA1* (-5.40±2.19) and *htrA2* (-3.86 ± 1.49) appeared to be downregulated in the corresponding mutant strain while the *cssRS* two-component system and the other two *htrA*-like proteases remain unaltered.

**Fig 3:**
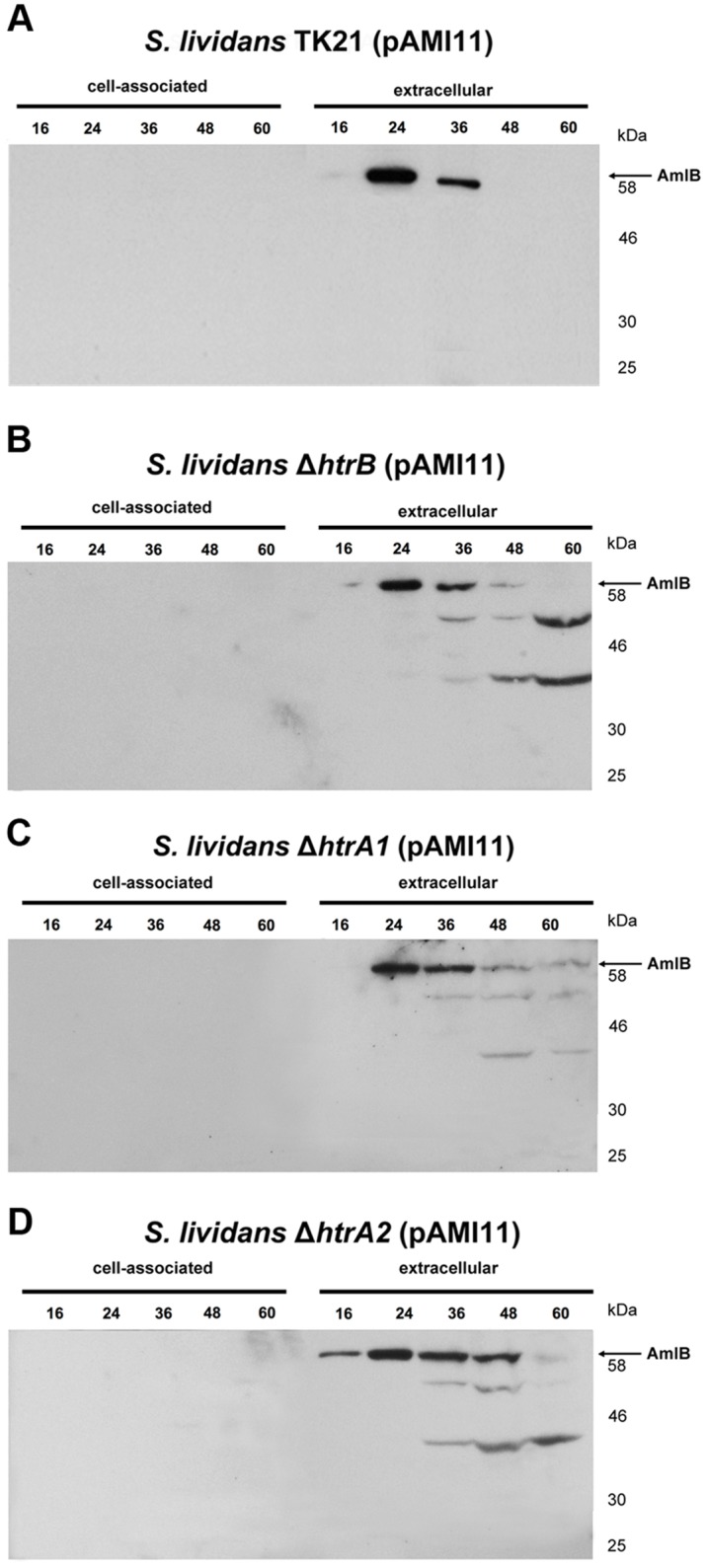
Alpha-amylase produced by *S*. *lividans* TK21(pAMI11), *S*. *lividans* Δ*htrB* (pAMI11), *S*. *lividans* Δ*htrA1* (pAMI11) and *S*. *lividans* Δ*htrA2* (pAMI11). Cell-associated and extracellular amylase present in *S*. *lividans* TK21 (pAMI11) (A), *S*. *lividans* Δ*htrB* (pAMI11) (B), *S*. *lividans* Δ*htrA1* (pAMI11) (C) and *S*. *lividans* Δ*htrA2* (pAMI11) (D) were analysed at different times of growth (16, 24, 36, 48 and 60 h) by Western blot using antibodies raised against AmlB. The amount of protein loaded onto the gels was corrected by the dried weight of the bacterial cultures. Molecular size markers are indicated on the side of each panel. The arrow indicates the relative mobility of the mature AmlB.

To study the role of the three HtrA-like proteases in the secretion stress response, the multicopy plasmid pAMI11 harbouring the alpha-amylase coding gene *amlB*, was propagated in the mutant strains. The growth rate of the HtrA-like proteases deficient strains overproducing alpha-amylase was slightly reduced when compared to that of the wild type overexpressing AmlB (not shown). AmlB was observed extracellularly in all cases when anti-AmlB serum was used in Western blot analyses. No pre-AmlB was detected in the different cellular fractions (Fig 3), as it occurs in the corresponding isogenic strains carrying plasmid pIJ486 not containing the *amlB* gene (not shown). The higher level of secreted alpha-amylase was observed in the exponential phase of growth in the HtrA-like protease deficient strains (Fig 3B–3D), where the enzyme degradation pattern was similar to the previously observed when the CssS- and CssR- deficient strains were analysed [3].

The activity of the secreted enzyme was measured and compared to that of the alpha-amylase produced by the wild type strain. The activity of the alpha-amylase secreted in the mutant strains was severely reduced (by 74%-85%) in comparison to that of the wild type. The decrease in the measured activity reflects the existence of misfolded secreted AmlB in each of the three HtrA-like protease deficient strains. This strongly suggests that the three proteases seem to be needed simultaneously in a functional manner to degrade the accumulated misfolded proteins.

### Overexpression of the HtrA-like genes

**Fig 4:**
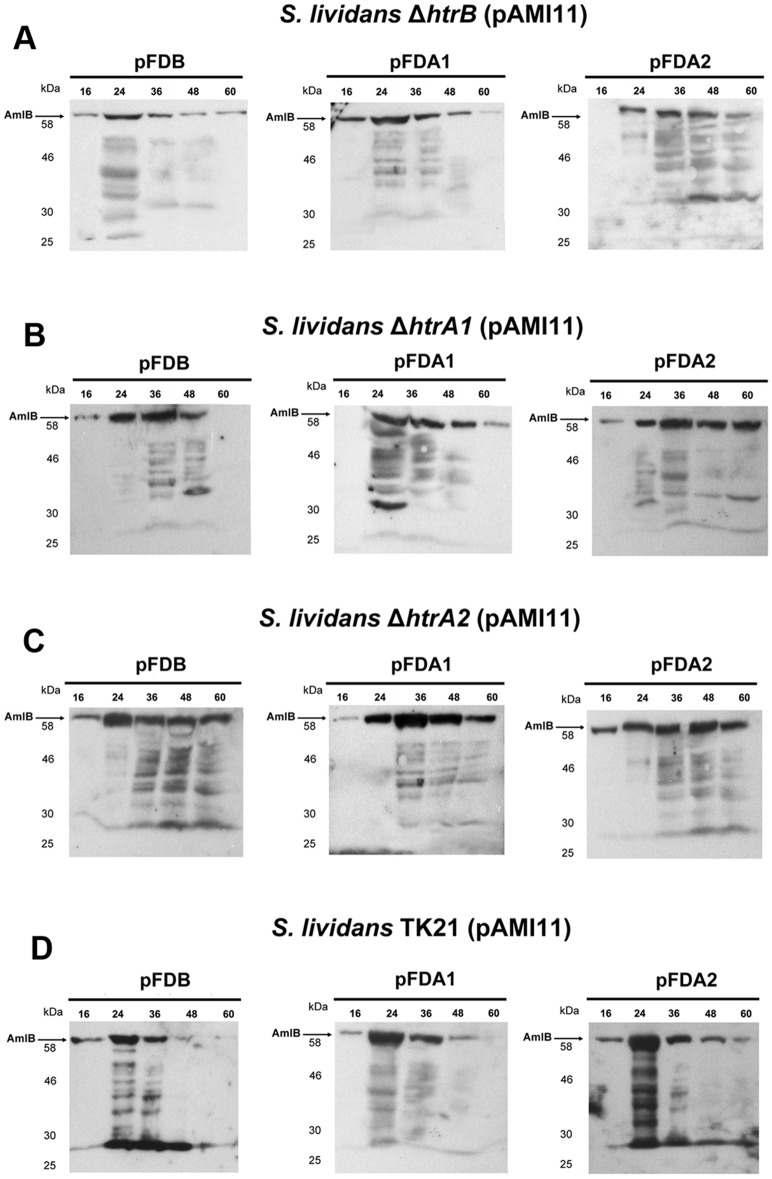
Complementation of the alpha-amylase synthesised by the HtrB, HtrA1 and HtrA2 deficient strains by propagation in multicopy of their respective genes. (A) Extracellular alpha-amylase present in the supernatants of the HtrB-deficient strain transformed with pFD666 derivative plasmids containing *htrB*, *htrA1* and *htrA2* (pFDB, pFDA1 and pFDA2, respectively) (B) HtrA1-deficient strain transformed with pFDB, pFDA1 and pFDA2 (C), HtrA2-deficient strain transformed with pFDB, pFDA1 and pFDA2 (D) and *S*. *lividans* TK21 transformed with pFDB, pFDA1 and pFDA2 were analysed at different times of growth (16, 24, 36, 48 and 60 h) by Western blotting assays using antibodies raised against AmlB. The amount of protein loaded onto the gels was corrected by the dried weight of the bacterial cultures. Molecular size markers are indicated on the side of each panel. The arrows indicate the relative mobility of the mature AmlB.

To ascertain if self- and cross-complementation of each HtrA-like deficiency could take place, the different HtrA-like coding genes were propagated in multicopy plasmids compatible with the plasmid carrying the alpha-amylase gene pAMI11, as described in Materials and Methods, and alpha-amylase production analysed by Western blot assays. A similar pattern was observed in all cases consisting of numerous bands of lower molecular size than those predicted for the mature alpha-amylase (Fig 4A–4C). This pattern was different to the one observed when the secreted alpha-amylase was analysed in each of the protease-deficient strains (Fig 3B–3D). This could be attributed to an imbalance in the secretion stress response resulting from the overexpression of the proteases in the bacterium. Propagation of the multicopy plasmids harbouring the different HtrA-like coding genes in *S*. *lividans* (pAMI11) produced a similar pattern to that observed when the different proteases were overexpressed in the mutant strains (Fig 4D), strongly suggesting that the overexpression of each protease may increase alpha-amylase degradation. This is in accordance with the severely reduction (up to a 94%) of alpha-amylase activity measured in the supernatants of the strains overexpressing each protease.

### Complementation of the HtrA-like protease deficient strains with their respective genes propagated in monocopy

Plasmids pSETB, pSETA1 and pSETA2 containing single copies of *htrB*, *htrA1* and *htrA2* respectively, were used to transform the HtrB-, HtrA1- and HtrA2-deficient strains carrying the alpha-amylase gene in multicopy to test if the homologous or heterologous single-copy complementation of the HtrB, HtrA1 and HtrA2 deficiency could take place. Alpha-amylase activity was restored exclusively when the HtrB-, HtrA1- and HtrA2-deficient strains were complemented by the *htrB*, *htrA1*, *htrA2* genes respectively (30%-81% of that of the wild type in the same conditions). No alpha-amylase activity was restored by cross-complementation by any of the other two HtrA-like coding genes, thus showing that no cross-complementation of the HtrA-like proteases can take place in *S*. *lividans*

## Discussion

HtrA-like proteases are widely distributed in nature, from bacteria to humans [33]. It has been shown in a wide range of bacterial pathogen species that HtrA proteases are essential for virulence and survival under environmental stress [33, 34]. The HtrA-like proteases perform essential functions acting as protein quality controllers while avoiding the accumulation of misfolded proteins in the periplasmic space.

Members of the HtrA-family are serine proteases with an Asp-His-Ser catalytic triad, where the aspartate and histidine residues increase the nucleophilicity of the serine hydroxyl group that hydrolyses peptide amide bonds. Their catalytic activity is strictly controlled and can be reversibly switched on and off, which does not occur in the case of classic Ser proteases. Additionally, the HtrA-family contains PDZ domains in the C-terminal half involved in protein-protein interactions, substrate recognition and/or regulation of protease activity [35]. HtrA proteins normally assemble into complex oligomers. Membrane-anchored HtrA-like proteases are active as trimers, and soluble HtrA-like proteases form larger active oligomers.

The number of paralogous HtrA-like proteins changes between different species and a significant number of bacterial genomes encode more than one HtrA-like protease [36], hence it is interesting to study the function of each paralogue and its implication in the protein quality control. In our work we characterise the three HtrA-like proteases previously identified in the *S*. *lividans* TK21 genome [3].

The phosphorylated regulator CssR in *B*. *subtilis* activates two HtrA-like proteases [4]. In *S*. *lividans* the phosphorylated regulator CssR binds to 302, 549 and 535 bp long DNA fragments containing the putative regulatory regions of *htrB*, *htrA1* and *htrA2*, respectively. The phosphorylated regulator binds to its own operon regulatory region, suggesting that the CssR protein directly regulates the operon *cssRS* as it occurs with the respective regulators of the *E*.*coli cpxAR* [30, 37] and the *B*. *subtilis cssRS* [4]. The “in silico” identification of the C(C/G)AGCT(G/T)CG motif was used to search into the *Streptomyces lividans* genome. The search revealed the presence of this motif in 348 different entries (not shown). This is probably due to the high content G+C of the *S*. *lividans* genome (72.24%), [20]. Therefore, the real involvement, if any, of this motif in the CssR transcriptional regulation remains to be determined.

Heterologous alpha-amylase production at the *B*. *subtilis* transition phase of growth induces the synthesis of the two HtrA-like proteases (HtrB and HtrA) of this bacterium: self-regulation and reciprocal cross-regulation occurs in *B*. *subtilis* in such a way that the expression of *htrB* and *htrA* is negatively regulated both by its own gene product and by the product of the other protease gene [36].

When individual mutations in each of the three HtrA-like proteases identified in *S*. *lividans* TK21 were analysed, the overproduced alpha-amylase detected in the corresponding supernatants presented a different pattern to that of the wild type strain, and very similar to that of the CssR-or CssS-deficient strains [3]. The pattern of the extracellular alpha-amylase observed in the Western blot analyses was consistent with the alpha-amylase being incorrectly folded, resulting on the appearance of lower molecular size bands that are absent in the wild type supernatant, which is probably the result of alpha-amylase degradation by other proteases present extracellularly. The extracellular alpha-amylase activity detected in the mutant strains was significantly reduced when compared to that of the wild type strain, suggesting the need for the three proteases to be in their fully functional form for the correct cleavage of misfolded proteins.

Interestingly though, the overexpression of each HtrA-like protease in any of the deficient strains and in the wild type strain results in a distortion in the quantity and quality of functional extracellular alpha-amylase (Fig 4), suggesting that the secretion stress response must be strictly balanced, where the presence in high copy number of any protease could negatively affect the mode of action of the others, as revealed by the pattern of degradation observed in the *S*. *lividans* TK21(pAMI11) oversynthesising some of the HtrA-like proteases.

These results, strongly suggest that to improve the overproduction of secretory proteins in *S*. *lividans*, the synthesis of the three HtrA-like proteases needs to be properly balanced to avoid negative effects in the secreted protein.

Complementation of each of the HtrA-like deficient strains by propagation of its own functional gene in single copy restored the extracellular alpha-amylase activity. This complementation did not take place when any of the other two HtrA-like encoding genes were propagated in the same manner, suggesting, once again, the need for a balanced, coordinated role of the three proteases to properly cleave the secreted misfolded alpha-amylase.

**Fig 5:**
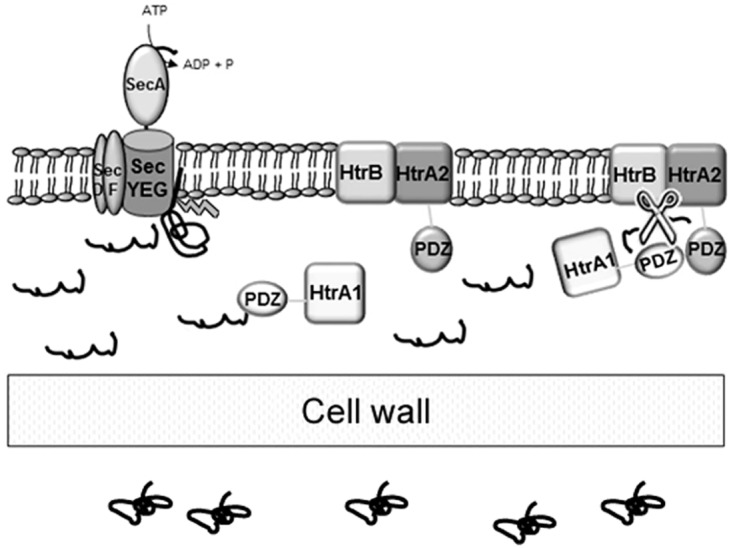
Predicted mode of action of the three HtrA-like proteases in *S*. *lividans*. HtrA1 recognizes the misfolded protein outside of the cytoplasmic membrane while the complex formed by HtrB and HtrA2 remain localised in the cytoplasmic membrane. The HtrA1 PDZ domain could interact with the HtrA2 PDZ domain turning the three proteases into an active state favouring the cleavage of the misfolded extracellular overproduced proteins.

A hypothetical mode of action of the three HtrA-like proteases in *S*. *lividans* is depicted in Fig 5. The analysis of the HtrA1 amino acid sequence revealed that it has a predicted signal peptide of 32 residues, a catalytic domain and a unique C-terminal PDZ domain. HtrB and HtrA2 have an N-terminal domain with a predicted membrane-spanning segment (N_in_-C_out_), a catalytic protease domain and only in the case of HtrA2 a C-terminal PDZ domain. Therefore, HtrA1 is presumably located outside of the membrane due to the lack of a transmembrane domain and the presence of a signal peptide in a similar manner to the *E*. *coli* DegP and *B*. *subtilis* cleaved HtrA form [38, 7]. DegP in *E*. *coli* and HtrA *B*. *subtilis* could act as chaperones [8,7]; we hypothesise that in *S*. *lividans* HtrA1 acts as a chaperone, probably recognising small hydrophobic residues at the C terminus of the misfolded proteins via its PDZ domain [35], and transports the protein to a complex formed by HtrB and HtrA2, localised in the cytoplasmic membrane. In a subsequent step, the PDZ domains of HtrA1 and HtrA2 could interact in a similar manner as in happens in the DegP case [35], turning the three proteases into an active state by forming a kind of chamber where the three catalytic protease domains constitute a central core favouring the cleavage of the misfolded extracellular proteins. Once the peptides resulting from the cleavage are released, the subunits of the complex would be disassembled as described for DegP [39]. Further research is now being conducted to ascertain if this model is indeed correct.

## Supporting Information

S1 Fig Analysis of the expression of His_6_-CssR in *E*. *coli* strain BL21 (DE3). A) *E*.*coli* cells overexpressing His_6_-CssR were grown and processed as described in Material and methods. The supernatant (S) containing the cytosol fraction was loaded onto a chromatography column filled with a Cobalt-containing resin. The concentration of the (S) fraction loaded onto the SDS-PAGE was fifty times higher than that loaded onto the IPTG induce cells. The flow-through (F) contains the unbounded protein. The column was washed two times (W1,W2) before eluting the His_6_CssR with a buffer containing 150 mM imidazole (E1-E6). B) The cell lysates from *E*. *coli* containing pET28a His_6_-CssR (pRHIS) inducted and non-inducted by IPTG were analysed by Western blot analysis with antibodies against the His_6_ tag. The cell lysates from *E*. *coli* containing pET28a inducted and non-inducted by IPTG were used as negative control.
(TIF)

S1 Table Proteins identified by nano LC–MS/MS Triple Tof analysis in the purified His_6_-CssR. The Table indicates the number of Mascot protein score and the number of peptides identified for each protein by nano mass spectrometry analysis in the eluted fraction (E2. S1 Fig).
(DOCX)
