# Supplementary figures and images for: The Three Streptomyces lividans HtrA-Like Proteases Involved in the Secretion Stress Response Act in a Cooperative Manner
Source: PLoS One. 2016 Dec 15;11(12):e0168112. doi: 10.1371/journal.pone.0168112 (PMC5157995; doi:10.1371/journal.pone.0168112)

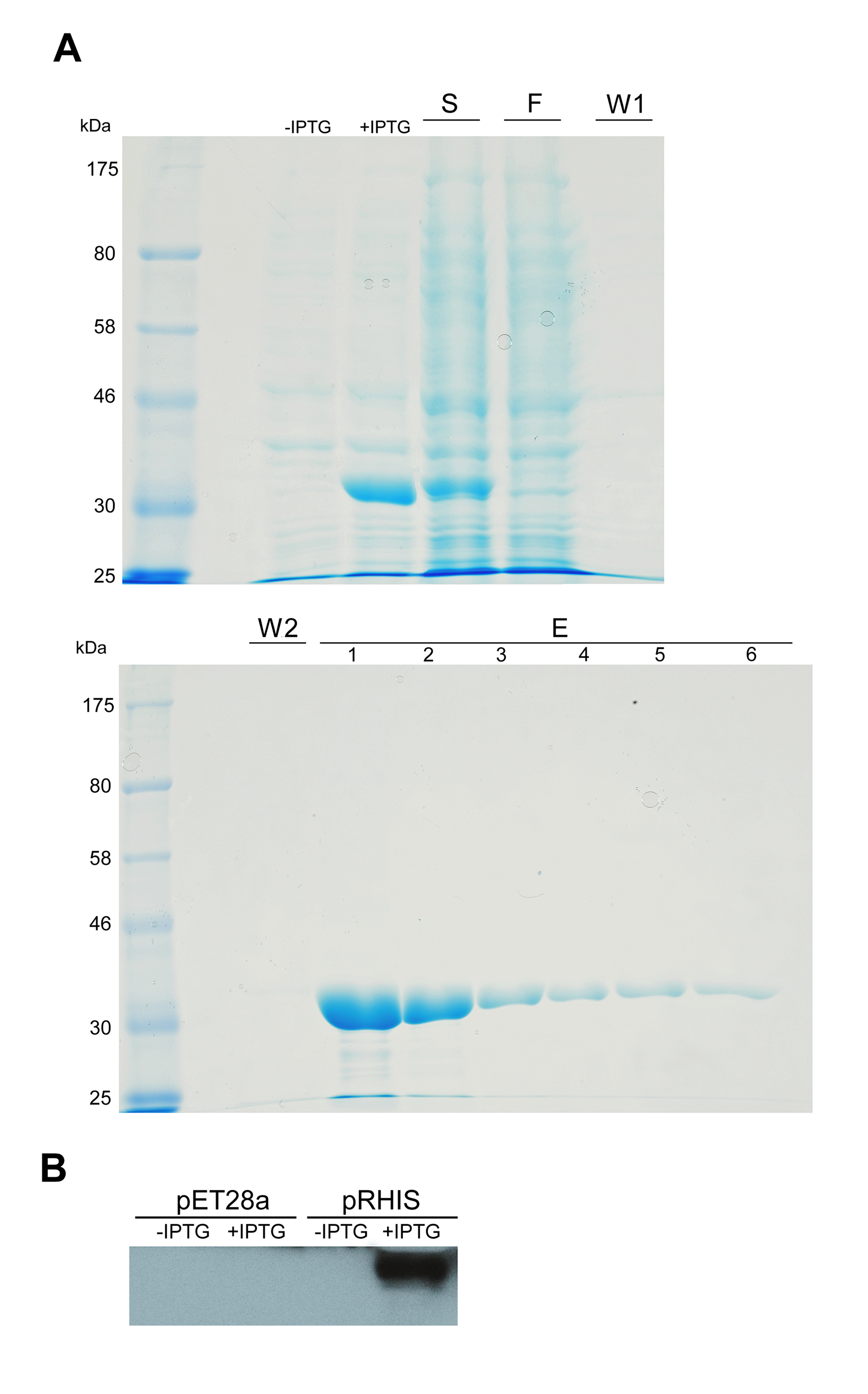

Supplement: S1 Fig — A) E.coli cells overexpressing His6-CssR were grown and processed as described in Material and methods. The supernatant (S) containing the cytosol fraction was loaded onto a chromatography column filled with a Cobalt-containing resin. The concentration of the (S) fraction loaded onto the SDS-PAGE was fifty times higher than that loaded onto the IPTG induce cells. The flow-through (F) contains the unbounded protein. The column was washed two times (W1,W2) before eluting the His6CssR with a buffer containing 150 mM imidazole (E1-E6). B) The cell lysates from E. coli containing pET28a His6-CssR (pRHIS) inducted and non-inducted by IPTG were analysed by Western blot analysis with antibodies against the His6 tag. The cell lysates from E. coli containing pET28a inducted and non-inducted by IPTG were used as negative control. (TIF) [file pone.0168112.s001.tif]
