# Supplementary material for: The Three Streptomyces lividans HtrA-Like Proteases Involved in the Secretion Stress Response Act in a Cooperative Manner
Source: PLoS One. 2016 Dec 15;11(12):e0168112. doi: 10.1371/journal.pone.0168112 (PMC5157995; doi:10.1371/journal.pone.0168112)
Supplement: S1 Table — The Table indicates the number of Mascot protein score and the number of peptides identified for each protein by nano mass spectrometry analysis in the eluted fraction (E2. S1 Fig). (DOCX) [file pone.0168112.s002.docx]

**Table S1. Proteins identified by nano LC–MS/MS Triple Tof analysis**

| Protein | Mascot protein score | Number of peptides identified |
| --- | --- | --- |
| CssR | 1483 | 56 |
| ArnA | 221 | 9 |
| Fur | 89 | 3 |
| RpsO | 75 | 4 |
| SlyD | 74 | 2 |
| RplM | 63 | 3 |

**in the purified His_6_-CssR.**

The table indicates Mascot protein score and the number of peptides identified for each protein by nano mass spectrometry analysis in the eluted fraction (E2. Fig S1).
